# Plant‐phenotypic changes induced by parasitoid ichnoviruses enhance the performance of both unparasitized and parasitized caterpillars

**DOI:** 10.1111/mec.16072

**Published:** 2021-07-20

**Authors:** Antonino Cusumano, Serge Urbach, Fabrice Legeai, Marc Ravallec, Marcel Dicke, Erik H. Poelman, Anne‐Nathalie Volkoff

**Affiliations:** ^1^ DGIMI Université de Montpellier INRAE Montpellier France; ^2^ Laboratory of Entomology Wageningen University Wageningen The Netherlands; ^3^ Department of Agricultural, Food and Forest Sciences University of Palermo Palermo Italy; ^4^ IGF Univ Montpellier CNRS INSERM Montpellier France; ^5^ BCM Univ Montpellier CNRS INSERM Montpellier France; ^6^ IGEPP Agrocampus Ouest INRAE Université de Rennes 1 Le Rheu France; ^7^ Université Rennes 1 INRIA CNRS IRISA Rennes France

**Keywords:** host‐parasitoid interaction, parasitoid‐associated symbionts, plant‐herbivore‐microbe interactions, plant‐mediated species interactions, polydnaviruses

## Abstract

There is increasing awareness that interactions between plants and insects can be mediated by microbial symbionts. Nonetheless, evidence showing that symbionts associated with organisms beyond the second trophic level affect plant‐insect interactions are restricted to a few cases belonging to parasitoid‐associated bracoviruses. Insect parasitoids harbour a wide array of symbionts which, like bracoviruses, can be injected into their herbivorous hosts to manipulate their physiology and behaviour. Yet, the function of these symbionts in plant‐based trophic webs remains largely overlooked. Here, we provide the first evidence of a parasitoid‐associated symbiont belonging to the group of ichnoviruses which affects the strength of plant‐insect interactions. A comparative proteomic analysis shows that, upon parasitoid injection of calyx fluid containing ichnovirus particles, the composition of salivary glands of caterpillars changes both qualitatively (presence of two viral‐encoded proteins) and quantitatively (abundance of several caterpillar‐resident enzymes, including elicitors such as glucose oxidase). In turn, plant phenotypic changes triggered by the altered composition of caterpillar oral secretions affect the performance of herbivores. Ichnovirus manipulation of plant responses to herbivory leads to benefits for their parasitoid partners in terms of reduced developmental time within the parasitized caterpillar. Interestingly, plant‐mediated ichnovirus‐induced effects also enhance the performances of unparasitized herbivores which in natural conditions may feed alongside parasitized ones. We discuss these findings in the context of ecological costs imposed to the plant by the viral symbiont of the parasitoid. Our results provide intriguing novel findings about the role played by carnivore‐associated symbionts on plant‐insect‐parasitoid systems and underline the importance of placing mutualistic associations in an ecological perspective.

## 1 INTRODUCTION

Plants are at the basis of most terrestrial food webs and interact with various organisms in nature, including herbivorous and carnivorous insects (Schoonhoven et al., 2005; Stam et al., 2014; Turlings & Erb, 2018). In recent years, there has been a rapidly growing body of evidence showing that plant‐insect interactions can be mediated by a large variety of microbial symbionts acting as “hidden” players (Mason et al., 2019; Pineda et al., 2017; Shikano et al., 2017). For instance, beneficial soil microbes such as plant growth‐promoting bacteria and fungi can enhance plant resistance against herbivore attack (Pineda et al., 2010; Pozo & Azcón‐Aguilar, 2007). On the other hand, microbes associated with herbivorous insects may help their hosts to exploit plants with unbalanced nutritional value (Douglas, 2015) or to counteract plant defences (Chung et al., 2013). Carnivorous organisms such as insect parasitoids also harbour a diversity of symbionts including bacteria, fungi and a wide array of viruses influencing plant‐insect interactions (Dicke et al., 2020). Yet, the effects of carnivore‐associated symbionts are far less investigated within a plant‐insect interaction perspective compared with plant‐ and herbivore‐associated symbionts (Cusumano & Volkoff, 2021).

Polydnaviruses are abundant and unique symbionts associated with larval endoparasitoids of the Ichneumonoidea (Braconidae and Ichneumonidae). Virus particles are produced exclusively in the calyx region of the ovary of parasitoid females from a proviral template. They are stored in the wasp oviducts and then injected by parasitoid females into a caterpillar host. Injection of polydnaviruses allows survival of parasitoid offspring within the herbivorous host by impairing host immune response and by altering host development and metabolism (Beckage, 2012; Burke & Strand, 2014; Lu et al., 2010; Strand et al., 2006; Webb et al., 2006). Two genera of polydnaviruses are defined, the Bracoviruses associated with braconid wasps and the Ichnoviruses associated with ichneumonid wasps (Francki et al., 1991).

To date, the only available information showing that polydnaviruses affect plant responses to herbivory is restricted to two plant‐herbivore parasitoid systems, both of which focused on bracoviruses (*Microplitis croceipes* bracovirus McBV, *Cotesia glomerata* bracovirus CgBV) (Cusumano et al., 2018; Tan et al., 2018; Zhu et al., 2018). Interestingly bracovirus manipulations in the caterpillars have been shown to change the composition of herbivore oral secretions (saliva and/or regurgitate), which often contain the elicitors exploited by plants to recognize insect damage (Bonaventure, 2012; Bonaventure et al., 2011; Rivera‐Vega et al., 2017). Indeed, the activity of glucose oxidase and β‐glucosidase is reduced in oral secretions of *Helicoverpa zea* and *Pieris brassicae* caterpillars injected with bracoviruses isolated from their respective endoparasitoid species, *M*. *croceipes* and *C*. *glomerata*. The consequence of bracovirus‐induced manipulations of caterpillar oral secretion is that plants downregulate defence‐related genes and reduce their chemical defences (Cusumano et al., 2018; Tan et al., 2018). In turn, bracovirus‐mediated changes in plant quality benefit the growth of the caterpillar in which the parasitoid larvae develop, thus increasing the fitness of the offspring of the bracovirus‐injecting parasitoid female (Tan et al., 2018). These discoveries have opened a novel scenario in plant‐insect interactions showing that, although third‐trophic level symbionts do not come directly in contact with the plant, they can still induce changes in plant phenotype, mediated by their effects on the infected caterpillars.

In addition to bracoviruses, other groups of parasitoid viruses are likely to affect plant‐insect interaction, since herbivory by parasitized caterpillars induces specific plant responses and parasitoid identity typically override the identity of the herbivore attacker (Cusumano et al., 2019; Poelman, Zheng et al., 2011; Zhu et al., 2015). We are only now starting to explore the diversity of parasitoid symbionts and the underlying mechanisms behind their interactions with the plants (Cusumano & Volkoff, 2021; Dicke et al., 2020; Shikano et al., 2017). From a molecular perspective, our understanding about the way parasitoid‐associated viruses manipulate caterpillar oral secretions is largely incomplete. Even though transcripts for a specific subset of bracoviruses genes have been detected in a host salivary gland (Bitra et al., 2011), it remains unclear if viral‐encoded proteins are actually present in caterpillar oral secretions. Furthermore, we are not aware of the full range of viral‐induced manipulations in caterpillar salivary glands, which caterpillar‐encoded proteins are upregulated and which ones are downregulated upon viral infection. The ecological effects that parasitoid‐associated viruses induce at the plant‐insect interface require further investigation as well. For example, an interesting but unexplored hypothesis is that other unparasitized herbivores also benefit from the increase in plant quality induced by parasitoid‐associated symbionts. The most common ecologically relevant scenario to test such hypothesis is to investigate whether unparasitized caterpillars take advantage from feeding on plants concurrently attacked by parasitized caterpillars (which thus are infected by parasitoid‐associated symbionts). In fact, parasitism levels in nature rarely reach 100% which means that usually not all herbivores feeding on a plant are parasitized.

The aim of the current study was to investigate for the first time whether ichnoviruses, which are associated with thousands of parasitoid species, affects the proteome of caterpillar oral secretions with cascading plant‐mediated interactions between parasitized and unparasitized caterpillars. Using as model species the solitary parasitoid *Hyposoter didymator* which carries *H*. *didymator* ichnovirus (HdIV), we investigated the role of ichnoviruses as hidden players in the interaction between corn (*Zea mais*) and the fall armyworm *Spodoptera frugiperda*. We experimentally manipulated the phenotype of herbivores by isolating calyx fluid (containing viral particles) from wasp females and injecting it into caterpillars subsequently feeding on corn plants. We specifically investigated: (1) whether ichnoviruses are responsible for the majority of the changes occurring in the salivary glands of naturally parasitized caterpillars by using a comparative proteomic approach, (2) whether the performance of unparasitized caterpillars increased when feeding on plants previously treated with insect saliva collected from caterpillars that had been injected with parasitoid calyx fluid containing the ichnovirus and (3) whether parasitoid offspring benefitted from plant‐mediated ichnovirus‐induced manipulation.

## 2 MATERIALS AND METHODS

### 2.1 Plants and insects

Corn plants (line B73 HT) were obtained from organic seeds at the Diascope experimental research station (INRA, Mauguio, France, 43°36'37"N, 3°58'35"E). Plants were grown in plastic pots (7 × 8 cm) in a climatic chamber at 25 ± 2°C, 60% RH and 16:8 h (L:D) and used for the experiments when they were two weeks old with four fully developed leaves. Plants were allowed to acclimatize under laboratory conditions at the insect quarantine platform PIQ (University of Montpellier, DGIMI laboratory) 3–5 days before the experiments.

The corn strain of *Spodoptera frugiperda* was maintained at 24 ± 2°C, 65% RH and 16:8 h (L:D) on a semisynthetic corn‐based diet (Poitout et al.,^1972^). The parasitoid *Hyposoter didymator* was maintained on *S*. *frugiperda* larvae in the same abiotic conditions, using second to third instar larvae for parasitism. *S*. *frugiperda* is not naturally present in France and is considered as a quarantine pest. Consequently, experiments described hereafter were conducted in a confined environment at the DGIMI insect quarantine platform (PIQ).

### 2.2 Isolation of ichnovirus particles from *H. didymator* and injection into *S. frugiperda* caterpillars

Calyx fluid (containing the ichnovirus particles) was extracted from *H*. *didymator* wasps anaesthetized on ice and dissected in phosphate‐buffered saline (PBS, 1x, pH 7.4 Fischer Life Technology) under a light microscope. The calyx region of the ovaries was collected in 250 µl PCR tubes. The volume was adjusted with PBS to reach the desired concentration in female equivalents (f.e.) as described in Dorémus et al. (2013) (for example, ovaries from 30 wasps pooled in 60 µl of PBS for injection of 100 nl containing 0.05 f.e./caterpillar). A concentration of 0.05 f.e. was selected based on preliminary investigations that showed a consistent effect on the phenotype of injected caterpillars (i.e., a reduction in the weight of injected caterpillars two days post injection compared to PBS‐injected caterpillars, Doremus et al., 2013). Calyx tissues were disrupted by several passages through a 20 µl micropipette cone. Tubes containing the disrupted biological material were centrifuged for 5 min at 5000 rpm and then supernatant containing the calyx extracts was stored on ice until injections into third instar *S. frugiperda* caterpillars were carried out (as described below). Viral purification by centrifugation has been shown to have similar effects on caterpillar physiology as other purification techniques such as filtration or using a gradient (Beckage et al., 1994). Presence of viral particles in calyx extracts was confirmed by negative staining and observation under an electron microscope Zeiss EM10CR at 80 kV.

Third instar (L3) *S*. *frugiperda* caterpillars anaesthetized on ice were injected using the Eppendorf FemtoJet 4x injector equipped with glass capillaries (3.5", Drummond Scientific. no. 3‐000‐203‐G/X) in order to prepare the following treatments: (1) “CF”: injection of calyx fluid containing ichnovirus particles dissolved in 100 nl of PBS, (2) “PBS”: unparasitized caterpillars injected with 100 nl of PBS (negative control), and (3) “PAR” caterpillars parasitized by *H*. *didymator* and injected with 100 nl of PBS (positive control). Parasitism by *H*. *didymator* was performed 2–4 h before injection with PBS. Parasitism was obtained by introducing individual *S*. *frugiperda* L3 larvae into a cylindric plastic container (diameter =15 cm, height =7.5 cm) with 10 *H*. *didymator* female wasps. The host larva was removed immediately after being stung once. Treatments 2 and 3 were used as controls to test whether the microinjection treatment per se has an effect on the subsequent investigations (i.e., interaction of the caterpillars with the food plant, changes in protein profile of salivary glands). Preliminary observations comparing parasitized caterpillars with caterpillars that, in addition to being parasitized, were also injected with PBS indicated no apparent effects of PBS injection on parasitoid development inside the caterpillar host. Injections with venom extracts were not carried out as previous experiments have shown that the venom does not play any apparent role in parasitism success of *H*. *didymator* on *S*. *frugiperda*, nor does the venom synergize the effect of ichnoviruses (Dorémus et al., 2013).

After microinjections, the caterpillars that recovered within 1 h were allowed to feed on corn plants for two days before using them for experiments (proteomic investigations of caterpillar salivary glands and performance bioassays). This time window was selected because two days post injection (p.i.) the parasitoid progeny in parasitized caterpillars is still at the egg stage, so it is possible to exclude any physiological effect on caterpillar phenotype due to the feeding by wasp larvae.

### 2.3 Salivary gland dissection, SDS‐PAGE and quantitative proteomic analyses

To investigate whether different injection treatments affect the full protein composition of caterpillar salivary glands, we carried out comparative proteomic investigations. Labial salivary glands from two days p.i caterpillars (treatments CF, PBS and PAR) were processed as described by Celorio‐Mancera et al. (2012). Briefly, glands were first dissected in cold PBS, then rinsed with cold PBS and placed in a droplet of 20 µl of cold PBS per pair of glands on a Petri dish kept on ice. Glands were cut in half inside the droplet, and subsequently transferred along with the buffer solution to 1.5 ml Eppendorf tubes. Glands of five individuals were pooled as a biological replicate. The samples were centrifuged for 5 min at 17,530 *g* at 4°C. The supernatant was transferred to new Eppendorf tubes, and protein concentration in the samples was quantified by Bradford spectrophotometric assay (Bradford, 1976). A preliminary assessment of the complexity of the salivary protein profiles between PAR‐, PBS‐ and CF‐treated caterpillars was obtained by separating the proteins using 4%–12% SDS‐PAGE precast mini gels (Biorad). Lanes were loaded with 20 µg of proteins in 20 µl in Laemmli buffer (62.5 mM Tris‐HCl pH 6.8, 2% SDS, 10% glycerol, 5% beta mercaptoethanol and 0.005% bromophenol blue). Electrophoresis was performed in 25 mM Tris‐HCl pH 8.8, 195 mM glycine, and 0.1% (w/v) SDS at a constant current of 35 mA. Gels were stained with colloidal blue (protein staining solution, Euromedex).

For the purpose of quantitative proteomic analyses, another SDS‐PAGE gel was prepared at the Functional Proteomics Platform (IGF BCM ‐ CNRS INSERM, Montpellier). Samples (20 µg of proteins in 40 µl in Laemmli buffer) were loaded onto the gel. After short migration, a unique portion of the gel which contained all bands was excised. Proteins were reduced, alkylated (with 10 mM DTT for 45 min at 56°C and 55 mM iodoacetamide for 30 min at room temperature, respectively), and then digested in‐gel using trypsin (1 µg, mass spectrometry grade, Promega), as previously described (Shevchenko et al., 1996).

Samples were analysed online using a nanoESI Qexactive HFX mass spectrometer (Thermo Fisher Scientific) coupled with an Ultimate 3000 HPLC (Thermo Fisher Scientific). Desalting and preconcentration of samples were performed on‐line on a Pepmap precolumn (0.3 × 10 mm). A gradient consisting of 0–25% B in 100 min, 25%–40% B in 20 min, 40%–90% B in 5 min (A = 0.1% formic acid, 2% acetonitrile in water; B = 0.1% formic acid in acetonitrile) at 300 nl/min was used to elute peptides from the capillary (0.075 × 150 mm) reverse‐phase column (Pepmap, Thermo Fisher Scientific). Nano‐ESI was performed with a spray voltage of 2 kV, and heated capillary temperature of 270°C. A cycle of one full‐scan mass spectrum (375–1500 m/z) at a resolution of 120,000, followed by 20 data‐dependent MS/MS spectra, was repeated continuously throughout the nanoLC separation. All MS/MS spectra were recorded using normalized collision energy of 28 at a resolution of 30,000 and an isolation window of 1.6 m/z.

Analysis was performed using maxquant software (Cox & Mann, 2008; version 1.6.10.43). All MS/MS spectra were searched using Andromeda (Cox et al., 2011) against a protein database consisting of forward and reverse translations from *S*. *frugiperda* genome (https://bipaa.genouest.org/sp/spodoptera_frugiperda_pub/), HdIV entries (Legeai et al., 2020) and 250 classical contaminants (maxquant contaminants database, http://www.maxquant.org/downloads.htm). Search parameters were default parameters with slight modification. Briefly, first search precursor mass tolerance was set to 20 ppm, and main search was set (after recalibration) to 6 ppm. A maximum of two missed‐cleavages was allowed. Search was performed allowing variable modifications: Oxidation (Met), Acetyl (Nterm) and with one fixed modification: Carbamidomethyl (Cys). False discovery rate (FDR) was set to 0.01 for peptide and proteins, and minimal peptide length to seven. Quantification was also performed using maxquant with standard parameters. Graphical representation and statistical analysis were done using perseus (Tyanova et al., 2016, v1.6.10.43) using standard workflow (reverse and contaminant entries removing, filtering based on number of valid value: at least three in one group, and then imputation using “Replace missing values from normal distribution” tool from perseus). All *t* tests were performed using a FDR of 5% and s0 of 0.1 (Tusher et al., 2001).

### 2.4 Performance bioassays

#### 2.4.1 Performance of parasitized and unparasitized caterpillars

To evaluate if plant induction with salivary gland extracts from differently injected caterpillars (PAR, CF or PBS) affects the performance of the herbivores, we carried out a relative growth rate experiment. Using salivary gland extracts for plant induction treatments allows to carefully control the amount of leaf damage which could affect plant responses. In fact, *S*. *frugiperda* caterpillars infected with HdIV (either parasitized or injected with calyx fluid) inflict significant less feeding damage to corn leaves compared to PBS‐injected caterpillars (see Supporting information, and Figure S3).

Each plant was damaged on the first fully expanded leaf using a pattern wheel. The wheel was rolled over the leaf surface on each side of the midrib, two lines in parallel (length 3 cm distance between each other of 0.75 cm) creating a c. 2.25 cm^2^ area with 20 tiny holes (~0.5 mm^2^). A total of 20 µl of salivary gland extract, prepared as described before, from differently injected *S*. *frugiperda* caterpillars was applied to the tiny holes on these mechanically damaged leaves. There were two groups of third instar caterpillars (parasitized or unparasitized) which were feeding on (1) undamaged plant leaves (UD) or leaves from plants treated with salivary gland extract from (2) PBS‐injected unparasitized caterpillars (PBS); (3) PBS‐injected parasitized caterpillars (PAR); (4) CF‐injected caterpillars (which are infected with HdIV) (CF) for a total of eight experimental combinations. Within each treatment, 16–18 successful replicates were carried out. The bioassays were performed in blocks, with the parasitism status of the feeding herbivore as the block factor. Treatments were randomized within each block.

Twenty‐four hours after application of salivary gland extract, the treated leaf was collected for the caterpillar feeding bioassay. Parasitized and unparasitized caterpillars were weighed and then fed on the treated corn leaves in plastic tubes closed with cotton wool and lined with 2% agar to keep leaves moist. Forty‐eight hours later, caterpillars were reweighed and relative growth rate was calculated as follows: (final weight − initial weight)/(average weight).

#### 2.4.2 Performance of parasitoids

To determine if the performance of parasitized caterpillars also influences development of the endoparasitoids, we conducted a parasitoid performance experiment. Third instar *S*. *frugiperda* caterpillars were parasitized by *H*. *didymator* and fed leaves from plants treated with salivary gland extract from PBS‐injected unparasitized caterpillars (PBS) or CF‐injected caterpillars (which are infected with HdIV) as described above. Twenty‐four hours after treatment, the treated leaf was collected and placed in a plastic tube closed with cotton wool and lined with 2% agar to keep leaves moist. Treated leaves were replaced every other day to keep food fresh until parasitoid cocoon formation. To assess the performance of the wasps we recorded: (1) developmental time (time from wasp parasitization until cocoon formation), (2) developmental mortality (proportion of wasps that yield a cocoon out of the total number of parasitized caterpillars), and (3) cocoon weight (recorded the second day after its formation).

### 2.5 Statistical analysis

A principal component analysis (PCA) comparing the proteomic profile of salivary glands from the three different caterpillar treatments was performed using log2 protein intensity in the perseus bioinformatics platform, using standard parameters. Differences in terms of protein abundances among the three caterpillar groups were investigated by performing a standard *t* test analysis in perseus (Tyanova et al., 2016). Relative growth rate data were transformed when needed to meet assumption of normality and homoscedasticity and analysed with ANOVA. Developmental time data of parasitoids were analysed with a general linear model (GLM) with gamma error distribution and inverse link function. Mortality data were analysed with a GLM with binomial error distribution and logit link function. Cocoon weight data were normally distributed and analysed with linear models. Significance of the factors in the GLMs was assessed using likelihood ratio tests (LRTs) (Crawley, 2007). Significance levels for factors in the linear model were derived directly from *F*‐tests. The adequacy of the statistical models was assessed with residual plots (Crawley, 2007). ANOVA and GLMs have been carried out with r statistical software (R Development Core Team, 2013).

## 3 RESULTS

### 3.1 Ichnovirus infection induced changes in the proteome of *S. frugiperda* salivary glands

**FIGURE 1:**
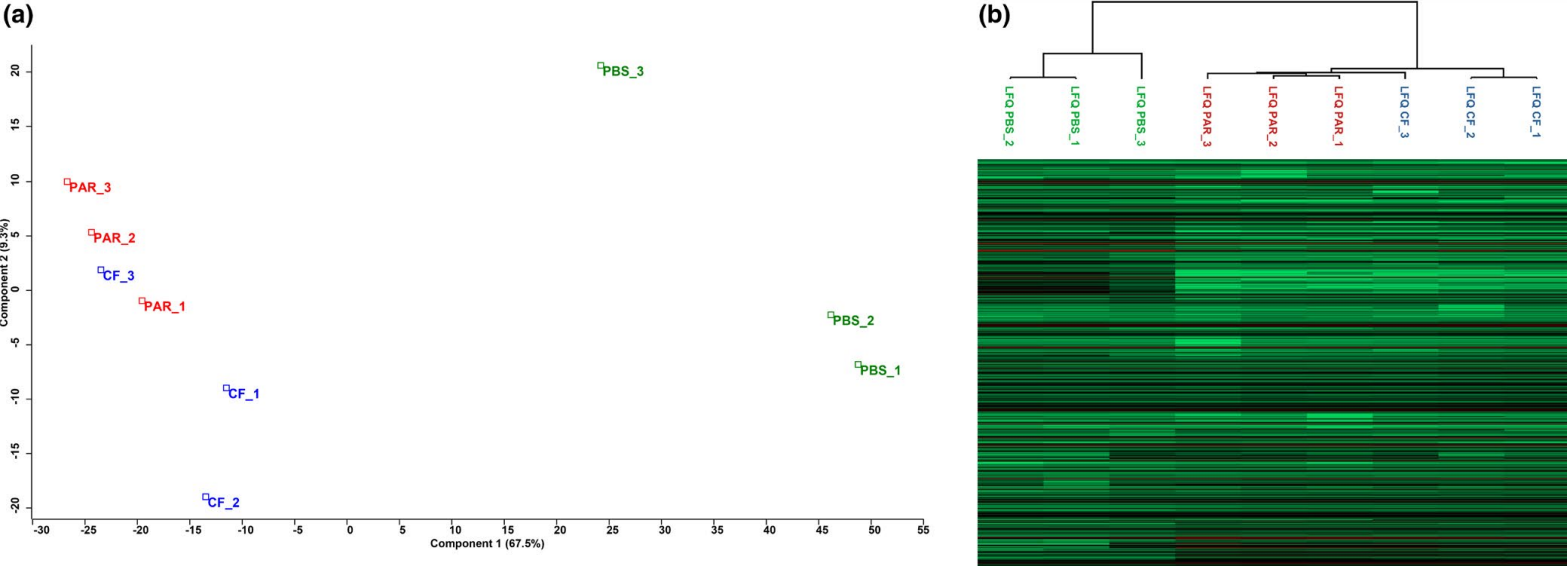
(a) Principal component analyses (PCA) based on proteins (LFQ = log2 protein intensity) detected in the salivary glands of the different caterpillar treatments. Blue squares: CF = *Spodoptera frugiperda* caterpillars injected with calix fluid (containing HdIV virions) isolated from the parasitoid *Hyposoter didymator*; Red squares: PAR = *S. frugiperda* caterpillars parasitized by *H*. *didymator*; Green squares: PBS = *S. frugiperda* caterpillars injected with phosphate‐buffered saline. (b) Hierarchical clustering based on Pearson correlation (same treatments and colour scheme as above)

Principal component analysis of the protein composition of salivary glands showed that PBS‐injected caterpillar samples clustered separately from the two other treatments, that is, parasitized and CF‐injected caterpillars. The first principal component explained 67.5% of the variation whereas the second component explained 9.3% of the variation. The distinction between CF and PAR samples is less pronounced but these groups can nonetheless be discriminated (Figure 1a). In a similar way, hierarchical clustering allows to separate the three treatments, with the control samples (PBS) clustering apart from CF and PAR samples (Figure 1b). There was a positive correlation between proteome replicates within each treatment group and also between proteome compositions of the different caterpillar groups (Figure S1), with a higher correlation between CF and PAR samples compared with PBS samples (Pearson correlation factor ranging from 0.93 to 0.97).

**FIGURE 2:**
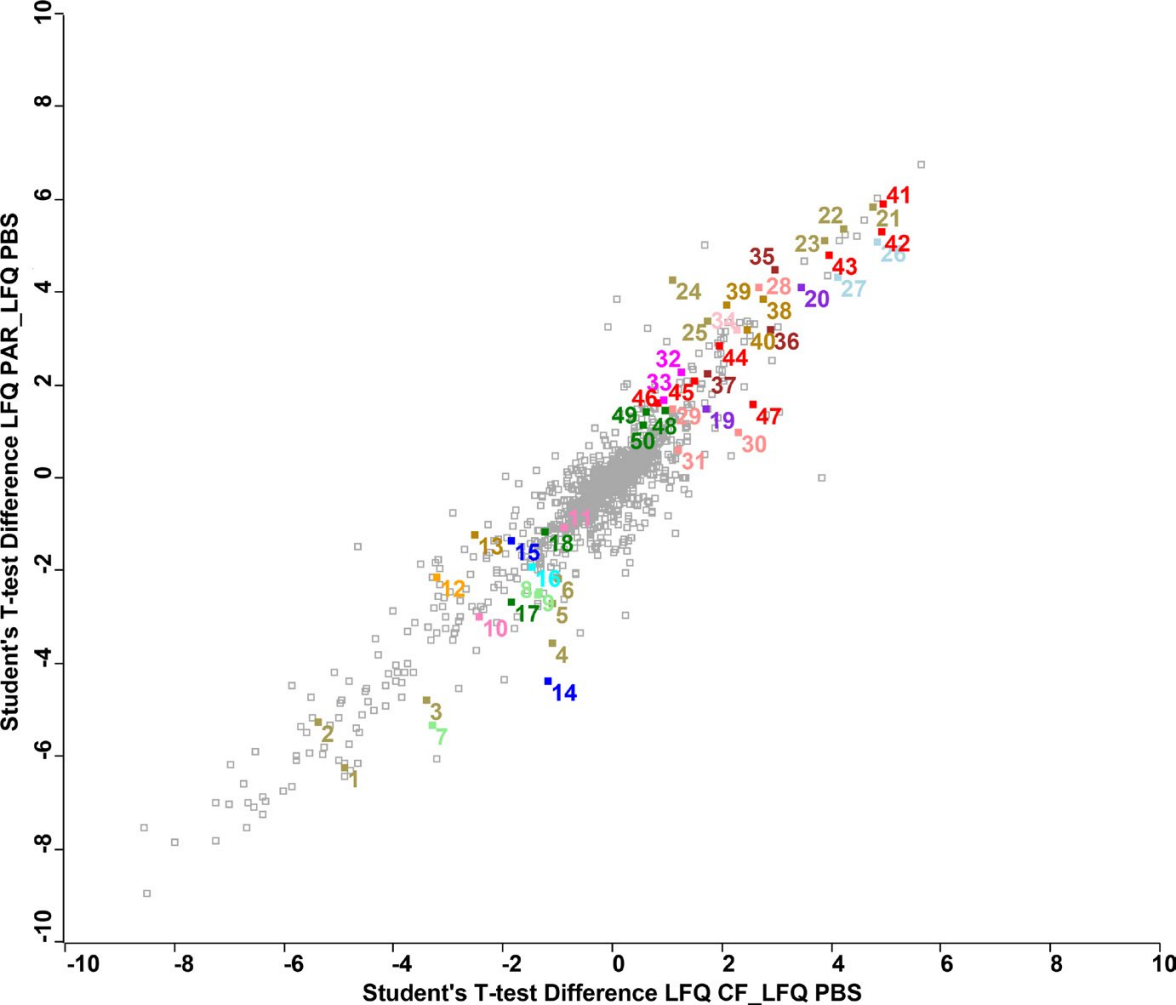
Scatterplot based on protein abundance ratio (LFQ = log2 protein intensity) detected in the salivary glands of the different *Spodoptera frugiperda* caterpillar treatments. CF = caterpillars injected with calix fluid (containing HdIV virions) isolated from the parasitoid *Hyposoter didymator*; PAR = caterpillars parasitized by *H*. *didymator*; PBS = caterpillars injected with phosphate‐buffered saline. Each square in the figures represents one protein detected in the salivary glands. The y‐axis of shows the Student's *t* test difference for each protein based on pairwise comparisons between PAR vs. PBS treatment. The x‐axis of plot shows the same value for CF vs. PBS treatment. Protein from Table 1 are highlighted (see number in first column), colours correspond to different families of proteins

**FIGURE 3:**
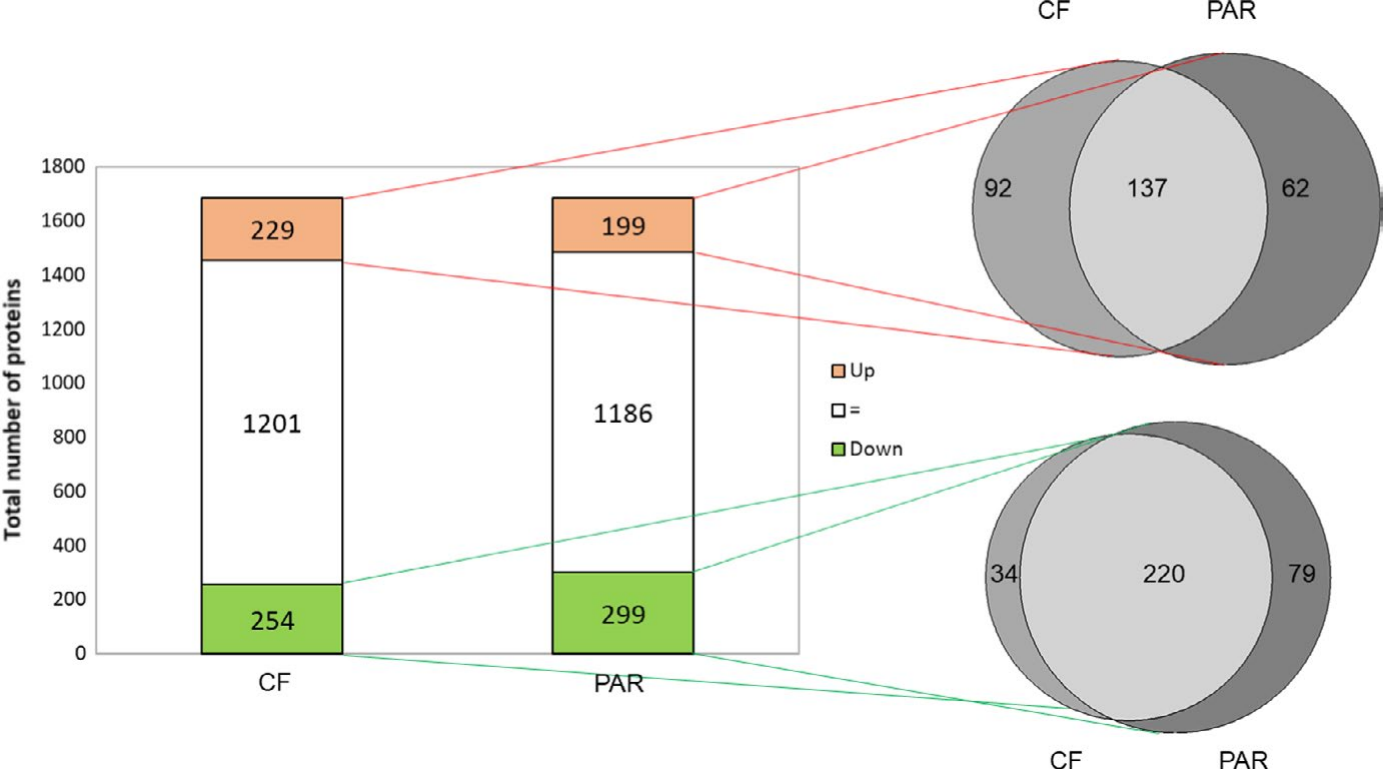
(Left) Total number of proteins found in salivary glands of *Spodoptera frugiperda* caterpillars: bars represent proteins significantly more abundant (red), less abundant (green) or not significantly different (white) in the virus‐infected (CF) and parasitized (PAR) treatments compared with saline‐injected controls (PBS). (Right) direct comparisons of the proteomic changes between CF and PAR treatments in the subset of proteins that displayed a significant increase in abundance (top venn diagram) or a significant decrease in abundance (lower venn diagram) in the previous comparison with PBS. Light grey colour indicates proteins shared in the CF and PAR treatments; medium grey colour indicates unique proteins of CF treatment; dark grey colour unique proteins of the PAR treatment

Compared to PBS‐injected caterpillars, protein abundance in PAR or CF treated groups is highly similar (Pearson correlation: 0.93, Figure 2). Globally, 1684 proteins were identified and quantified in this analysis. A total of 624 proteins (37%) display differences in abundance in CF and PAR treated groups compared to PBS‐injected control caterpillars: 291 proteins (17%) are significantly more abundant in the treatment groups, and 333 (20%) proteins are more abundant in the PBS group (Figure 3). Most of the proteins affected are common to both CF and PAR conditions (220 out of 333 for those less abundant, and 137 out of 291). As expected from the high correlation between PAR and CF samples (Figure S1), only minor variations were observed between these two treatments: all protein entries except one (GSSPFG00005192001‐PA, Table S1) had levels similarly affected in either PAR or CF samples (Figure S2).

### 3.2 Nature of *S. frugiperda* salivary gland proteins affected by parasitism or HdIV infection

Out of the 624 proteins differentially expressed by either parasitism or calyx fluid injection, a subset of 335 *S*. *frugiperda* entries corresponded to proteins with levels significantly affected with at least a two‐fold change (log2Dif>|1|). From that, 112 proteins were more abundant in saliva from PAR‐ or HdIV‐infected caterpillars and 223 proteins were more abundant in saliva from PBS‐injected control caterpillars (Table S1). Among the latter, 29% (65 entries out of 223) corresponded to ribosomal proteins. Ribosomal proteins are cellular proteins and are not a priori components of the saliva. However, this finding indicates that the protein synthesis machinery may be downregulated in salivary glands from CF‐infected caterpillars. Thereafter, we focused on the proteins that harboured a predicted signal peptide in their available sequence suggesting that the corresponding proteins are secreted and thus potentially present in *S. frugiperda* saliva where they could impact plant response. The set of potentially secreted proteins included 18 downregulated entries and 32 upregulated entries differentially represented in treated samples (PAR or CF) compared to controls (PBS) (Table 1). To this list of potential effectors in insect‐plant interactions, we also added an entry corresponding to glucose oxidase (GOX; GSSPFG00008369001‐PA), which despite the absence of a predicted signal peptide, is described in the literature as a major lepidopteran salivary protein and a herbivory‐associated elicitor (Chen & Mao, 2020; Rivera‐Vega et al., 2017).

**TABLE 1:** List of the subset of proteins detected in the salivary glands of *Spodoptera frugiperda* caterpillars with decreased (green) or increased (red) abundance in the presence of HdIV virions (i.e., both treatments with calyx fluid‐injected and parasitized caterpillars) compared to saline‐injected controls (PBS). Proteins shown in this list have a cutoff score in terms of intensity (log2 LFQ differences) >|1| and a predicted signal peptide. Numbers ahead of protein IDs refer to numbers indicated in Figure 2

Protein IDs	Description BLAST2GO	SP: predicted signal peptide	Peptide counts (all)	LFQ difference significant (+)			log2 LFQ difference			Peptides	Unique peptides	Sequence coverage [%]	Identified in FAW saliva (Acevedo et al., 2017)	Putative biological function
				PAR_PBS	CF_PBS	CF_PAR	PAR_PBS	CF_PBS	CF_PAR					
Proteins with decreased levels in presence of HdIV														
Hydrolases/carboxylesterases														
1. GSSPFG00001832001.3‐PA;GSSPFG00026973001.2‐PA;GSSPFG00014816001.3‐PA	Juvenile hormone esterase‐like (Spodoptera litura)	SP	61;1;1	+	+		−6.24342	−4.89666	1.34676	61	3	79	yes	Hydrolase activity
2. GSSPFG00003779001.5‐PA	Juvenile hormone esterase‐like (Spodoptera litura)	SP	62	+	+		−5.27334	−5.3682	−0.094864	62	4	79	yes	Hydrolase activity
3. GSSPFG00035209001.5‐PA;GSSPFG00003781001.2‐PA;GSSPFG00035206001.3‐PA	Juvenile hormone esterase‐like (Spodoptera litura)	SP	40;38;1	+	+		−4.78173	−3.39309	1.38863	40	38	77.2	yes	Hydrolase activity
4. GSSPFG00001834001.5‐PA	Carboxyl choline esterase cce016d (JHE‐like)	SP	25	+			−3.57053	−1.102	2.46853	25	7	52.6	yes	Carboxypeptidase activity
5. GSSPFG00003780001.2‐PA	Juvenile hormone esterase‐like (Spodoptera litura)	?	25	+			−2.71133	−1.09253	1.6188	25	5	61.9	yes	Hydrolase activity
6. GSSPFG00003782001.3‐PA	Antennal esterase cxe10 (JHE like)	SP	28		+		−2.17156	−0.987827	1.18373	28	10	56.6	no	Hydrolase activity
Oxidases and oxidoreductases														
7. GSSPFG00008369001‐PA	Glucose oxidase**	NO	20	+	+		−5.32304	−3.30184	2.0212	20	20	59	yes	Glucose oxidase activity
8. GSSPFG00022903001‐PA	Peroxidase‐like (Spodoptera litura)	SP	49	+	+		−2.53264	−1.36211	1.17053	49	31	59.6	yes	Peroxidase activity
9. GSSPFG00007595001‐PA;GSSPFG00006078001‐PA;GSSPFG00006079001‐PA;GSSPFG00003386001‐PA	Peroxidase‐like (Spodoptera litura)	SP	22;1;1;1	+	+		−2.46694	−1.33673	1.13021	22	4	70.7	yes	Peroxidase activity
Transferases														
10. GSSPFG00033338001.3‐PA;GSSPFG00035238001.3‐PA	UDP‐glucuronosyltransferase 2B20‐like	SP	6;1	+	+		−3.00742	−2.44122	0	6	6	37.1	no	Transferase activity
11. GSSPFG00000775001.1‐PA	Choline‐phosphate cytidylyltransferase a‐like isoform x1	SP	4	+	+		−1.08306	−0.874714	0.208342	4	4	14.1	no	Transferase activity
Isomerases														
12. GSSPFG00030721001‐PA	Protein disulphide‐isomerase A6 homologue (Spodoptera litura)	SP	4	+	+		−2.15341	−3.20195	0	4	4	14.2	yes	Protein disulphide isomerase activity
Serine protease inhibitors														
13. GSSPFG00032953001‐PA	Serine protease inhibitor‐like (Spodoptera litura)	SP	5	+	+		−1.23126	−2.52576	0	5	5	16.5	no	Serpin
Calcium binding proteins														
14. GSSPFG00030086001.1‐PA	Protein eyes shut (Spodoptera litura)	SP	2	+	+		−4.37739	−1.17179	3.2056	2	2	1.1	no	Calcium ion binding
15. GSSPFG00002987001‐PA;GSSPFG00032257001‐PA	Fibulin 1	SP	3;1		+		−1.34618	−1.8347	−0.488521	3	2	2.9	no	Calcium ion binding
Matrix proteins														
16. GSSPFG00019005001‐PA	Sparc	SP	12	+	+		−1.93518	−1.46528	0.469896	12	12	48.5	no	Extracellular matrix glycoprotein
Uncharacterized proteins														
17. GSSPFG00003200001.1‐PA	Uncharacterized protein LOC111347839	SP	4	+	+		−2.69189	−1.83861	0	4	4	31	na	Unknown
18. GSSPFG00019412001‐PA	Uncharacterized protein LOC111350498	SP?	26	+	+		−1.15692	−1.22626	−0.06934	26	26	76.8	na	Unknown
** No predicted signal peptide														
Proteins with increased levels in presence of HdIV														
Viral proteins														
19. GlyPro1_Hd2	GlyPro1_Hd2	SP	5	+	+		1.47395	1.72178	0.247836	5	4	18.4	na	Viral protein
20. GlyPro2_Hd2	GlyPro2_Hd2	SP	10;4	+	+		4.11443	3.4537	−0.660737	10	9	42.2	na	Viral protein
Hydrolases/carboxylesterases														
21. GSSPFG00019678001.3‐PA	Bile salt‐activated lipase‐like	SP	16	+	+		5.8274	4.77259	−1.05481	16	16	40.1	no	Hydrolase activity
22. GSSPFG00017956001‐PA	Pancreatic triacylglycerol lipase‐like	SP	6	+	+		5.37689	4.21194	−1.16495	6	5	36.7	no	Hydrolase activity
23. GSSPFG00008537001‐PA	Pancreatic lipase‐related protein 2‐like	?	7	+	+		5.12243	3.86411	−1.25832	7	6	42.2	no	Hydrolase activity
24. GSSPFG00023328001‐PA	Lipase 3‐like [Spodoptera litura]	SP	7	+			4.2679	1.08743	−3.18047	7	7	28.8	no	Hydrolase activity
25. GSSPFG00005453001‐PA	Pancreatic triacylglycerol lipase‐like	SP	18	+	+		3.3682	1.72996	−1.63824	18	16	56.6	no	Hydrolase activity
Hydrolases/sulphatases														
26. GSSPFG00018527001‐PA	Sulphatase B [Spodoptera frugiperda]	SP	18	+	+		5.07659	4.83222	−0.244371	18	3	34.7	no	Hydrolase activity
27. GSSPFG00033998001‐PA	Sulphatase B [Spodoptera frugiperda]	SP	21	+	+		4.3316	4.11084	−0.220762	21	6	38.7	no	Hydrolase activity
Glycoside hydrolases														
28. GSSPFG00031044001‐PA	Chitinase‐like protein EN03 [Spodoptera litura]	?	19	+	+		4.11302	2.67403	−1.43899	19	19	71.1	no	Hydrolase activity
29. GSSPFG00031440001.1‐PA	Myrosinase 1‐like	SP	7	+			1.49512	1.10719	−0.387939	7	7	16.3	no	Hydrolase activity
30. GSSPFG00010046001‐PA	Alpha‐l‐fucosidase‐like	SP	5		+		0.968729	2.28932	1.32059	5	5	11.9	no	Hydrolase activity
31. GSSPFG00027172001.1‐PA;GSSPFG00025461001‐PA	Alpha‐amylase 2	SP	12;6		+		0.586316	1.19842	0.612109	12	12	14.5	no	Hydrolase activity
Hydrolases other														
32. GSSPFG00024259001‐PA;GSSPFG00025402001.1‐PA	Glycerophosphoryl diester periplasmic	SP	7;1	+			2.25735	1.25706	−1.00028	7	7	28.3	no	Hydrolase activity
33. GSSPFG00024543001.1‐PA	Uncharacterized protein LOC111352027	SP	8	+	+		1.67183	0.945632	−0.726201	8	8	24.6	no	Hydrolase activity
Nucleotidases														
34. GSSPFG00034710001‐PA;GSSPFG00009246001‐PA	Apyrase	SP	24;2	+	+		3.19762	2.27136	−0.926257	24	2	56.4	yes	Hydrolase activity
Oxidoreductases														
35. GSSPFG00020128001.3‐PA	Glucose dehydrogenase (FAD, quinone) like (Helicoverpa armigera)	SP	26	+	+		4.48165	2.95524	−1.52641	26	26	59.9	yes	Oxidoreductase activity
36. GSSPFG00021013001.3‐PA;GSSPFG00012369001‐PA	Prophenoloxidase subunit 1	SP?	13;5	+	+		3.17882	2.90057	−0.278256	13	13	22.6	yes	Oxidoreductase activity
37. GSSPFG00013976001.3‐PA	Prophenoloxidase subunit 2	SP?	10	+	+		2.23837	1.72614	−0.512234	10	10	15.9	yes	Oxidoreductase activity
Serine protease inhibitors														
38. GSSPFG00030018001‐PA	Zonadhesin‐ partial	SP	23	+			3.83281	2.74237	−1.09044	23	19	23.8	no	Serpin
39. GSSPFG00027636001‐PA	Zonadhesin‐ partial	?	9	+			3.70744	2.08892	−1.61852	9	5	17.3	no	Serpin
40. GSSPFG00007350001‐PA	Interalpha‐trypsin inhibitor heavy chain H4‐like	SP	24	+			3.20066	2.47077	−0.729885	24	24	32.1	no	
Others														
41. GSSPFG00026146001‐PA	Mucin‐17‐like [Helicoverpa armigera]	SP	34	+			5.90745	4.95865	−0.948792	34	31	16.5	no	Structural protein
42. GSSPFG00025266001‐PA	Fibrohexamerin‐like [Spodoptera litura]	SP	12	+	+		5.30111	4.93287	−0.368245	12	12	60.4	no	Silk protein
43. GSSPFG00032545001.3‐PA	MD‐2‐related lipid‐recognition protein‐like	SP	7	+	+		4.79631	3.94988	−0.84643	7	7	68	no	Sterol transport protein
44. GSSPFG00013557001‐PA	Seroin transcript 1B [Spodoptera frugiperda]	SP	4	+	+		2.82901	1.9592	−0.869801	4	4	25.5	no	Silk protein
45. GSSPFG00015954001.4‐PA;GSSPFG00010998001.4‐PA	Small heat shock protein	SP?	4;4	+	+		2.0914	1.49402	−0.597378	4	4	18.5	no	HSP20‐like chaperone
46. GSSPFG00033956001‐PA	76.21_protein D2‐like	SP	8	+			1.59484	0.824924	−0.769917	8	8	33.8	no	Phosphatidylethanolamine‐binding protein
47. GSSPFG00032498001.3‐PA	Small heat shock protein 27.2	SP	6		+		1.58244	2.57465	0.992206	6	6	35.3	no	HSP20‐like chaperone
Uncharacterized proteins														
48. GSSPFG00019413001‐PA	Uncharacterized protein LOC111350556	SP	18	+			1.43937	0.962845	−0.476526	18	18	61.2	no	Unknown
49. GSSPFG00010771001.1‐PA	Uncharacterized protein LOC111351038	SP	7	+			1.41226	0.61264	−0.799621	7	7	53.4	no	Unknown
50. GSSPFG00019415001‐PA;GSSPFG00005645001‐PA	Uncharacterized protein LOC111350507	SP	24;24	+			1.12607	0.574136	−0.551933	24	24	78.8	no	Unknown
														

The proteome of salivary glands from parasitized or calyx fluid‐injected caterpillars contain two related viral proteins, GlyPro1 and GlyPro2, that both belong to the glycine and proline rich protein family. This finding strongly suggests that the salivary gland tissue is actually infected by the parasitoid symbiont HdIV. Among the proteins whose levels are increased in PAR and CF samples compared to PBS (Table 1), we found five putative lipases, several hydrolases, the two prophenoloxidases, and a nucleotidase (apyrase). Three serine protease inhibitors seem also affected by parasitism or calyx fluid injection as well as a mucin‐like protein and a fibrohexamerin‐like protein.

Conversely, proteins less abundant in PAR and CF samples include a number of enzymes belonging to various classes. The most strongly affected appear to be five different carboxylesterases with similarity with insect juvenile hormone esterase, a UDP‐glucuronosyltransferase, two entries matching with peroxidases, and a protein disulphide‐isomerase. GOX is also significantly less abundant in both PAR (log2Dif of −5.3) and CF (log2Dif of −3.3) samples compared to PBS controls (Figure 2).

### 3.3 Performance bioassays

#### 3.3.1 Performance of unparasitized caterpillars

**FIGURE 4:**
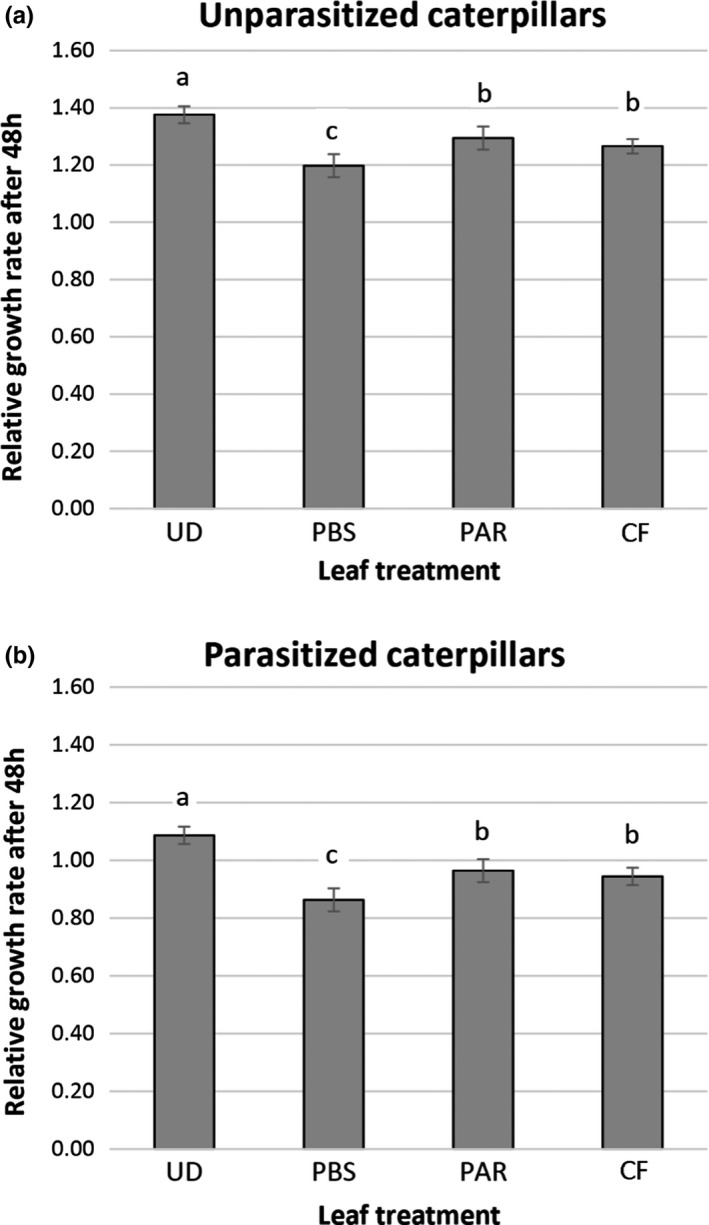
Relative growth rate of unparasitized (a) and parasitized (b) *Spodoptera frugiperda* caterpillars scored 48 h after feeding on corn leaves either undamaged (UD) or induced with salivary gland extract from: caterpillars injected with phosphate‐buffered saline (PBS); caterpillars parasitized by *Hyposoter didymator* (PAR); and caterpillars injected with calix fluid (containing virions) isolated from the parasitoid *H*. *didymator* (CF). Different letters above bars indicate significant differences among treatments (GLM, *p* < .05)

The relative growth rate of unparasitized *S*. *frugiperda* caterpillars was significantly affected by the leaves induced with different types of caterpillar saliva that were offered as food (ANOVA, *F* = 18.372, df = 3,63, *p* < .001). Caterpillars allowed to feed on leaves that were previously induced with saliva from unparasitized herbivores (treatment PBS) displayed reduced relative growth rates compared with caterpillars feeding on leaves induced with saliva from parasitized or virus‐infected caterpillars (treatments PAR and CF, respectively). No differences in relative growth rate were found when caterpillars were feeding on leaves previously induced with saliva obtained from either PAR or CF treated caterpillars (Figure 4a).

#### 3.3.2 Performance of parasitized caterpillars

Similarly, the growth rate of parasitized caterpillars was significantly affected by the leaves induced with different types of caterpillar saliva that was offered as food (ANOVA, *F* = 19.375, df = 3,64, *p* < .001). Parasitized caterpillars feeding on leaves previously induced with saliva from unparasitized caterpillars (treatment PBS) showed the lowest relative grow rates. Again, no differences were found in relative growth rates between caterpillars feeding on leaves previously induced with saliva from either parasitized or virus‐infected caterpillars (treatments PAR and CF, respectively) (Figure 4b).

#### 3.3.3 Performance of parasitoids

Parasitoid larvae developed significantly faster when their host caterpillars were feeding on leaves previously induced with salivary gland extract from CF‐injected caterpillars compared with PBS‐injected unparasitized caterpillars (χ^2^ = 14.744, df = 1, *p* < .001) (Table 2). In contrast, neither cocoon weight (*F* = 0.102, df = 1, 53, *p* = .751) (Table 2) nor parasitoid mortality (χ^2^ = 0.005, df = 1, *p* = .941) (Table 2) were affected by plant induction treatment.

**TABLE 2:** Performances of *Hyposoter didymator* parasitoids that developed into *Spodoptera frugiperda* caterpillars feeding on corn leaves previously induced with salivary gland extract from caterpillars injected with phosphate‐buffered saline (PBS) or caterpillars injected with calix fluid (CF) containing HdIV virions. Developmental time (days) is recorded from oviposition to cocoon formation; Cocoon weight (mg) is recorded the second day after its formation as fresh weight; developmental mortality (%) is calculated as the proportion of wasps that yield a cocoon out of the total number of parasitized caterpillars. For each performance determinant, different letters indicate significant differences between treatments (GLM, *p* < .05)

	PBS	CF
Developmental time (days)	7.89 ± 0.12^a^	7.26 ± 0.11^b^
Developmental mortality (%)	20.03 ± 0.51^a^	19.81 ± 0.45^a^
Cocoon weight (mg)	30.00 ± 7.34^a^	30.77 ± 8.49^a^

## 4 DISCUSSION

Insect parasitoids have been shown to interact with the plant by influencing plant responses to herbivory as a consequence of the parasitisation of the attacking herbivore (Cuny et al., 2019; Kaplan et al., 2016; Ode et al., 2016; Poelman, Gols et al., 2011; Poelman, Zheng et al., 2011; Tan et al., 2019, 2020). While it was previously assumed that parasitoid larvae growing within the herbivore body were responsible for the specific responses of plants to feeding by parasitized caterpillars (Poelman, Zheng et al., 2011), it is now acknowledged that parasitoid‐associated symbionts can be the real hidden driving forces mediating such complex interactions (Dicke et al., 2020; Shikano et al., 2017). Here, we report the first molecular and ecological evidence that ichnovirus infection affects plant‐insect interactions, increasing the awareness that such parasitoid‐associated symbionts have a much more extended phenotype than was previously thought.

From a mechanistic perspective, it has been hypothesized that parasitoid‐associated viruses could interact directly or indirectly with the plant (Cusumano & Volkoff, 2021). A direct interaction would occur when viral‐encoded proteins come in contact with the plant tissues. This is a fascinating hypothesis which is based on the evidence that bracovirus genes are expressed in salivary glands (Bitra et al., 2011; Zhu et al., 2018), suggesting that virus‐encoded proteins could be produced in the insect saliva and released into the plant during caterpillar feeding. Polydnaviruses could also act indirectly when viral injection in the caterpillar haemolymph induces physiological changes which alter the biochemical composition of caterpillar salivary glands. Evidence for the indirect mechanism of action is available for two bracoviruses, as targeted approaches have shown that the activity of enzymes known to activate plant defences is reduced after virus injection (Tan et al., 2018; Zhu et al., 2018).

Our results demonstrate that an ichnovirus may affect insect‐plant interactions both directly and indirectly. In our proteomic analyses, we found two virus‐encoded proteins present in salivary glands of *S*. *frugiperda* caterpillars naturally parasitized by *H*. *didymator* or injected with calyx fluid containing HdIV. Both are related glycine‐proline rich proteins, encoded by the same HdIV‐specific gene family, and known to be highly expressed and secreted in parasitized hosts (Volkoff et al., 1999). The presence of “alien” proteins in caterpillar salivary glands is indicative of qualitative changes that could be used by the plant to recognize whether the herbivore attacker has been parasitized or not and tailor the defences accordingly. There is increasing evidence showing that plants reduce their defences when attacked by caterpillars carrying polydnaviruses‐associated parasitoids (Cusumano et al., 2018; Tan et al., 2018); thus it is possible to argue that viral “alien” proteins, which represent reliable signatures of herbivory inflicted by parasitized caterpillars, could play a role in plant defence‐signalling pathways. Nonetheless, to confirm that such proteins come in contact with the plant, further analyses are needed to test if viral proteins can be detected in leaf tissue damaged by caterpillars infected with HdIV. If this holds true, the following step would be to test whether plants attenuate their defences when leaf tissues are induced with in vitro produced ichnoviral proteins.

Our quantitative analyses show that the protein profile of caterpillar salivary glands is strongly affected by ichnovirus infection (Table S1). Proteins involved in plant defence regulation such as GOX or apyrase are differently affected by the virus. GOX levels decreased in parasitized or virus‐infected caterpillars compared to PBS‐injected caterpillars, whereas apyrase levels increased. GOX has been shown to affect the strength of plant‐insect interactions in a plant‐specific manner: whereas this enzyme induces plant defences in tomato (Tian et al., 2012), it appears to suppress defences in tobacco (Musser et al., 2002) and its effect in corn remains unclear (Louis et al., 2013). In tomato, the bracovirus associated with *M*. *croceipes* (McBV) manipulates plant responses to herbivory by decreasing GOX activity (Tan et al., 2018). The ichnovirus associated with *H*. *didymator* (HdIV) could act in a similar way, although it remains to be investigated if performances of *S*. *frugiperda* caterpillars are affected by exogenous application of GOX in corn. Apyrase is an ATP‐hydrolyzing enzyme previously described in *S*. *frugiperda* (Acevedo et al., 2017) and *Helicoverpa zea* saliva (Wu et al., 2012). Application of *H*. *zea* apyrase to wounded tomato leaves was shown to downregulate plant defences (Wu et al., 2012). Although the effect of apyrase on corn still remains to be analysed, the observed increase in apyrase levels in HdIV‐infected *S*. *frugiperda* salivary glands may contribute to a decrease in plant defences induced by herbivory.

Our results also indicate that parasitism or virus injection affect different functional classes of proteins in *S*. *frugiperda* salivary glands including lipase‐like proteins, sulphatases B‐like salivary enzymes, juvenile hormone esterase, ecdysone oxidase and prophenoloxidase (see Supporting information for further discussion). However, whether these proteins are actually secreted in the gland duct and, if they are, whether they affect plant‐herbivore interactions remain to be determined. Furthermore, virus‐injection was able to reproduce most of the changes in expression levels occurring in naturally parasitized caterpillars. Considering that we sampled salivary glands from parasitized caterpillars before wasp egg hatching, our results corroborate the hypothesis that polydnaviruses are the major driver of the physiological changes induced in the insect host, whereas the parasitoid offspring itself seems to play a negligible role at this time of observation. Yet, future studies should investigate if polydnavirus‐induced changes in protein profile of caterpillar saliva are time‐specific and whether the role played by parasitoid larvae becomes progressively more important as they grow bigger inside the herbivores.

In turn, plant‐phenotypic changes triggered by the different composition of caterpillar oral secretions affect the performances of subsequent herbivores. We found indirect evidence that plant nutritional quality is increased after induction with saliva from ichnovirus‐infected herbivores, due to an increase in caterpillar performance when feeding on induced plant leaves. Recently, it has been shown that feeding of bracovirus‐infected caterpillars increased plant quality when compared to saline‐injected caterpillars by reducing activity of defence proteins such as polyphenol oxidase or trypsin inhibitor (Tan et al., 2018). It is well known that plant nutritional quality can indirectly impact parasitoid fitness via effects on the herbivore host (Ode, 2006). Interestingly we found an increase in performance not only for parasitized caterpillars but also for unparasitized caterpillars which thus take advantage of plant‐mediated ichnovirus‐induced manipulations. Considering that often in natural conditions not all herbivores feeding on the plants are parasitized, an increase in the performance of unparasitized herbivores represents a challenge for the plant and may suggest a top‐down ecological cost imposed by the parasitoid‐associated virus.

Ichnovirus‐manipulation of plant responses to herbivory leads to benefits for the parasitoid *H*. *didymator* in terms of reduced developmental time, although we find no evidence for other major fitness‐related proxies such as mortality or bodyweight. It is possible to argue that a faster development of parasitoids helps to escape from mortality risks due to natural enemies such as predators and hyperparasitoids. In particular hyperparasitoids are common fourth‐trophic level component of terrestrial trophic networks and they could strongly reduce the population densities of their parasitoid hosts (Cusumano et al., 2020; Sullivan & Völkl, 1999; Tougeron & Tena, 2019). Yet the hyperparasitoid complex of *H*. *didymator* is unknown and field experiments are required to test the hypothesis that ichnovirus‐induced plant manipulation leads to a reduction of hyperparasitism levels. Among the parasitoid‐associated symbionts, only another polydnavirus from the bracovirus family (McBV) has been shown to increase parasitoid performance via plant‐mediated effects (Tan et al., 2018). Interestingly the beneficial effects of McBV for the parasitoid *M*. *croceipes* are stronger compared with the effects of HdIV for the parasitoid *H*. *didymator* found in our study. Yet dissimilarities between the two tri‐trophic systems make comparisons challenging, especially at the plant level as on study focused on tomato‐bracovirus interactions and our study investigated corn‐ichnovirus interactions. Thus more experimental evidence is needed to conclude that bracoviruses achieve stronger plant‐mediated benefits for their symbiotic partners compared with ichoviruses.

Polydnaviruses are the most intensively studied mutualistic symbionts of parasitoids, yet research has generally been restricted to their role in host‐parasitoid interactions (Edson et al., 1981; Lu et al., 2010; Shelby & Webb, 1999; Strand et al., 2006; Strand & Burke, 2013; Webb et al., 2006). As a consequence, the effects of polydnaviruses on tissues such as hemocytes (involved in insect immunity) and fat bodies (involved in general metabolism) have been intensively studied while we know very little about the role played by such viruses in tissues like salivary glands and midgut which are important at the plant‐insect interface. Studies that will investigate the temporal patterns and tissue specificity of polydnavirus infection during the whole parasitoid development inside the herbivore host will be particularly informative for understanding how plant‐insect interactions are shaped by parasitoid symbionts. By extending the study of polydnaviruses at the plant level, novel positive and negative effects for their symbiotic parasitoid partners have been discovered. While some research has shown that top‐down manipulation of plant quality increases parasitoid fitness indirectly (Tan et al., 2018), other research has unraveled surprising ecological costs as well (Zhu et al., 2018). For example, polydnaviruses initiate an interaction network across four trophic levels which trigger changes in herbivore‐induced plant volatiles attracting insect hyperparasitoids (Zhu et al., 2018). Interestingly the extended phenotype of polydnaviruses can also reach other plant‐associated insects. If polydnaviruses enhance the performance of unparasitized herbivores feeding on the plant, as shown in this study, then there could be negative effects at the plant level. Future research should be undertaken in order to unravel what are the overall consequences of top‐down effects induced by parasitoid‐associated viruses for the plant fitness. Placing microbial mutualistic symbioses in a community context is thus crucial in order to fully understand the “hidden” role that polydnaviruses play in plant‐based food webs.

## CONFLICT OF INTEREST

The authors declare that the research was conducted in the absence of any commercial or financial relationships that could be construed as a potential conflict of interest.

## AUTHOR CONTRIBUTION

A.C., M.D., E.H.P. and A.‐N.V. conceived and designed the experiments. A.C., M.R. and S.U. performed the experiments. A.C., F.L. and S.U. analysed the data. A.C. wrote the first draft of the manuscript with input from S.U., M.D., E.H.P. and A.‐N.V.

## Supporting information

Supplementary Material

Table S1

Supplementary Material

## Data Availability

The data that supports the findings of this study have been made available in the Dryad Digital Repository at the following citation: Cusumano et al. (2021).
