# Supplementary material for: Plant‐phenotypic changes induced by parasitoid ichnoviruses enhance the performance of both unparasitized and parasitized caterpillars
Source: Mol Ecol. 2021 Jul 20;30(18):4567–83. doi: 10.1111/mec.16072 (PMC8518489; doi:10.1111/mec.16072)

**Supplemental Information for:**

**Plant-phenotypic changes induced by parasitoid ichnoviruses enhance the performance of both unparasitized and parasitized caterpillars**

A Cusumano^1,2,3^, S Urbach^4,5^, F Legeai^6,7^, M Ravallec^1^, M Dicke^2^, EH Poelman^2^ and A-N Volkoff^1^

**Salivary gland proteins**

A number of potentially secreted **lipase-like proteins** were found as more abundant in CF and PAR samples compared to PBS (Table 1). Lipases were described as potential plant elicitors in the grasshopper/*Arabidopsis* model where salivary lipases were shown to alter levels of herbivory-induced defense metabolites (Schafer et al 2011). Similarly, a **mucin-like protein** (GSSPFG00026146001-PA) is significantly more abundant in CF and PAR compared to PBS samples (Table 1). In our ichnovirus model, this protein could act as an elicitor of plant defense by entering into the plant cells through wounding sites, as described for the mucin like protein NIMLP produced and injected into plants by leafhoppers (Shangguan et al 2018). Among others insect-derived elicitors (reviewed in Rivera-Vega et al 2017, Chen and Mao, 2020), some were found in our dataset of 1684 identified proteins (e.g. glucosidase subunit B GSSPFG00011178001-PA, catalase GSSPFG00030477001.4-PA) but without significant differences between controls and CF or PAR samples (Supplementary Table 1). Finally, it is also possible that HdIV suppresses other unknown elicitors in caterpillar salivary glands which could attenuate plant-induced responses and chemical defenses.

Indeed, differences in protein abundance also concerned several enzymes secreted in *S. frugiperda* salivary glands. Amongst those significantly more abundant in CF and PAR compared to PBS samples, we can identify two **sulfatases B-like** enzymes (Table 1). Sulfatases hydrolyze sulfate esters of a wide range of substrates, including carbohydrates, steroids and proteins. *Plutella xylostella* utilizes a sulfatase to modify glucosinolates in order to suppress the defense in cruciferous host plants (Ratzka et al 2002), what could also be the case of *S. frugiperda*, a polyphagous insect occasionally reported on cruciferous plants (Casmuz et al 2010; CABI Invasive Species Compendium <https://www.cabi.org/isc/datasheet/29810>). A higher level of sulfatases in parasitized caterpillars could provide them an advantage when feeding on certain categories of host plants. On the other side, salivary enzymes such as a **UDP-glucuronosyltransferase**, a class of enzymes involved in detoxification process of plant allelochemicals (Ahmad et al 1993) appear reduced in CF-injected samples compared to controls (PBS). Interestingly, we also found differences in enzymes potentially related to insect hormone processing (Table 1). Several entries with similarity with the **juvenile hormone esterase**, an enzyme that hydrolyze juvenile hormone, appear as significantly less abundant in CF and PAR samples. Conversely, **ecdysone oxidase**, an enzyme that normally contributes to the synthesis of ecdysone, is more abundant in PAR and CF samples (Supplementary table 1); however, note that the available sequence does not harbor a signal peptide and the enzyme may not be secreted in the saliva. Finally, **two prophenoloxidase subunits**, which are involved in insect immune response, were also found more abundant in CF and PAR compared to PBS samples.

**References**

Ahmad SA, Hopkins TL: β-glucosylation of plant phenolics by phenol β-glucosyltransferase in larval tissues of the tobacco hornworm, *Manduca sexta* (L.). Insect Biochem Mol Biol. 1993, 23: 581-589.

CABI Invasive Species Compendium. Datasheet of *Spodopotera frugiperda*: <https://www.cabi.org/isc/datasheet/29810>

CASMUZ A, Juarez ML, Socias MG, Murua MG, PRIETO S, MEDINA S, WILLINK E, GASTAMINZA G. Review of the host plants of fall armyworm, *Spodoptera frugiperda* (Lepidoptera: Noctuidae). Revista de la Sociedad Entomológica Argentina. 2017 Mar 18;69(3-4).

Chen CY and Mao YB. Research advances in plant–insect molecular interaction. F1000Research 2020, 9(F1000 Faculty Rev):198. <https://doi.org/10.12688/f1000research.21502.1>

Rivera-Vega LJ, Acevedo FE, Felton GW. Genomics of Lepidoptera saliva reveals function in herbivory. Current opinion in insect science. 2017 Feb 1;19:61-9.

Schäfer M, Fischer C, Meldau S, et al.: Lipase activity in insect oral secretions mediates defense responses in Arabidopsis. Plant Physiol. 2011; 156(3): 1520–34.

Shangguan X, Zhang J, Liu B, Zhao Y, Wang H, Wang Z, Guo J, Rao W, Jing S, Guan W, Ma Y. A mucin-like protein of planthopper is required for feeding and induces immunity response in plants. Plant Physiology. 2018 Jan 1;176(1):552-65.

**Supplementary** **Figure 1** Heatmap based on the proteins (LFQ=log2 protein intensity) detected in caterpillar salivary glands performing a pairwise-comparisons between samples with Pearson correlation. Dots represent proteins. CF= *Spodoptera* *frugiperda* caterpillars injected with calix fluid (containing HdIV virions) isolated from the parasitoid *Hyposoter didymator*; PAR= *S.* *frugiperda* caterpillars parasitized by *H. didymator*; PBS= *S.* *frugiperda* caterpillars injected with phosphate-buffered saline.


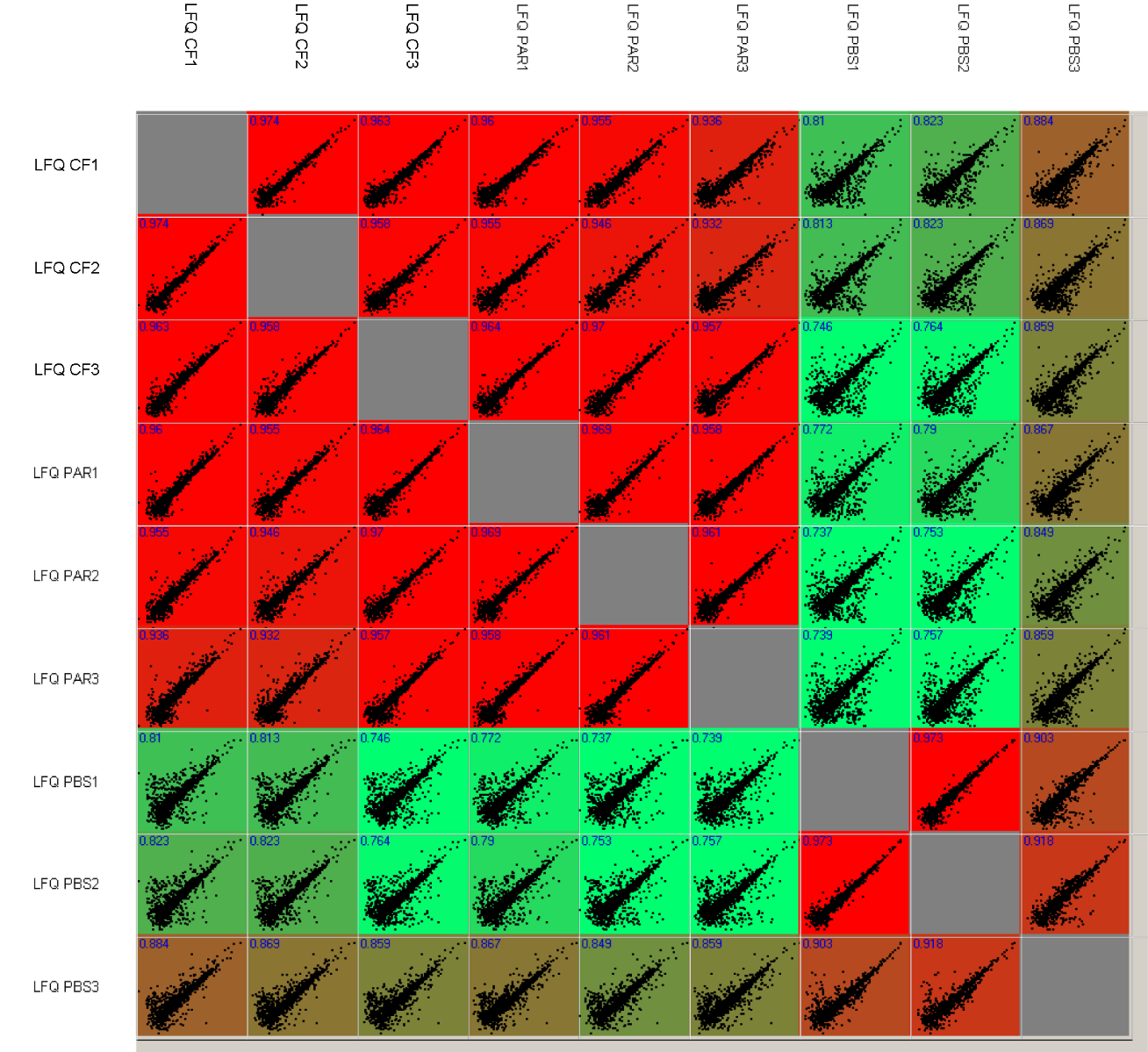


**Supplementary** **Figure 2**. Volcano plots based on proteins (LFQ=log2 protein intensity) detected in the salivary glands of the different *Spodoptera frugiperda* caterpillar treatments. CF = caterpillars injected with calix fluid (containing HdIV virions) isolated from the parasitoid *Hyposoter didymator*; PAR = caterpillars parasitized by *H. didymator*; PBS = caterpillars injected with phosphate-buffered saline. Each square in the figures represents one protein detected in the salivary glands. The y-axis of each plot shows the log10 p-value for each protein based on pairwise comparisons between caterpillar treatments (*t*-test). The x-axis of each plot shows the log2 fold change of each protein. Proteins found to be statistically significantly different (based on an FDR of 5% and s0 of 0.1) are highlighted in blue. A) Comparison between CF and PBS; B) Comparison between PAR and PBS; C) Comparison between PAR/CF.

A) CF / PBS


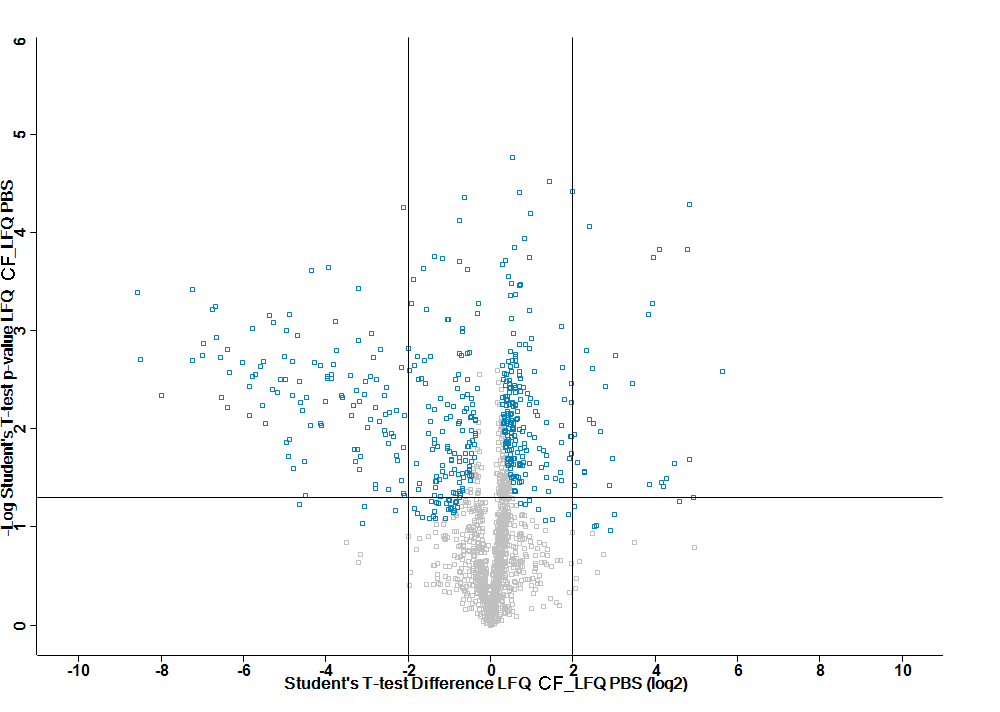


B) PAR/PBS


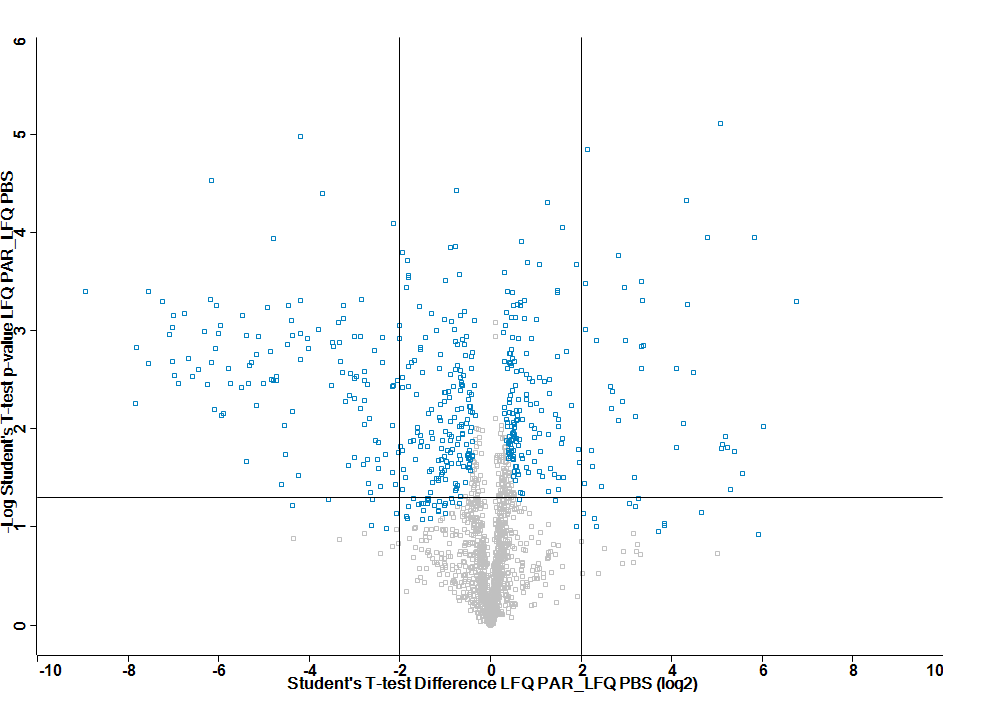


C) PAR/CF


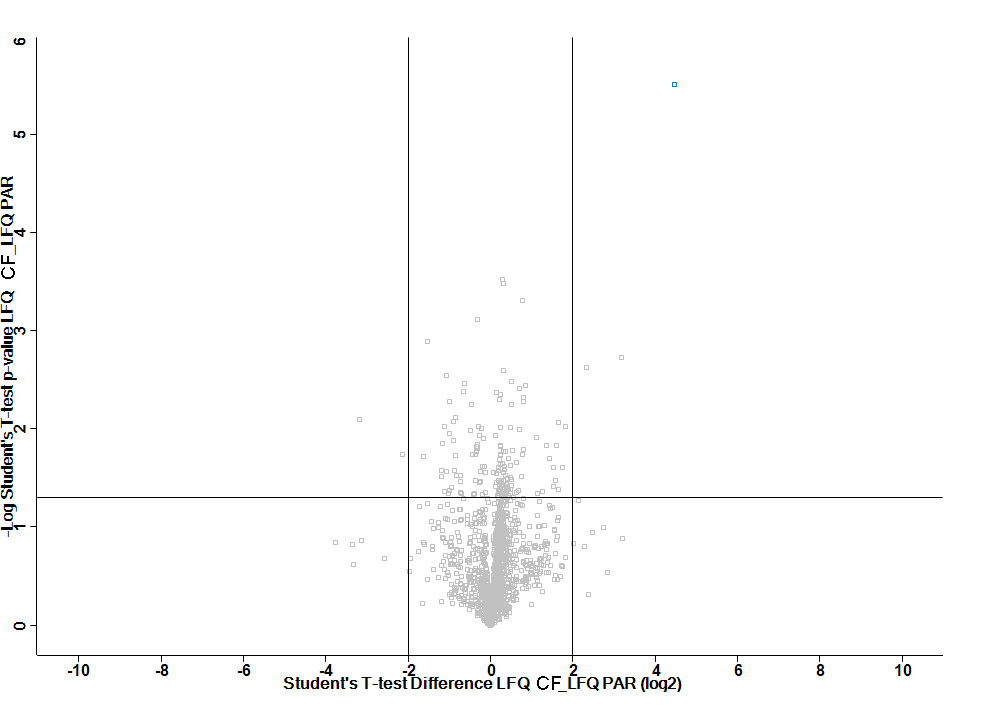


**Quantification of leaf area consumed by caterpillars**

To investigate whether differently injected *Spodoptera frugiperda* caterpillars inflict significantly different amount of damage to corn plants, we quantified the leaf area consumed by herbivores. Third instar (L3) *S. frugiperda* caterpillars were treated as described in the main text to obtain the following injection treatments: (1) “CF”: injection of calyx fluid from the parasitoid *Hyposoter didymator* containing ichnovirus particles dissolved in 100 nL of PBS; (2) “PBS”: unparasitized caterpillars injected with 100 nL of PBS; (3) “PAR” caterpillars parasitized by *H. didymator* and injected with 100 nL of PBS.

Injected caterpillars were allowed to recover for 1h and then they were transferred to corn plants with 4 fully developed leaves (line B73 HT). Each plant was enclosed together with 2 caterpillars with the aid of a nylon mesh bag (size = 30 cm × 40 cm; mesh count = 300 mesh/cm2). Caterpillars were allowed to feed on corn plants for 24 h. Subsequently insects were removed and leaves were cut to assess the amount of caterpillar feeding. The leaves were taped onto a white paper sheet (A4) and scanned with a RICOH Scanjet MPC4503. The scans were analyzed for the size of damaged leaf surface by counting the number of pixels making up the damaged area using Adobe Photoshop-CC 2015.1.2. The number of pixels was converted into cm^2^ by comparison with the number of pixels that make up a reference 1 cm^2^ square. We then used ANOVA to test the effect of the fixed factor “caterpillar treatment” on the response variable ‘leaf area damage’.

Results indicated that the injection treatment strongly affected the amount of herbivory inflicted to corn leaves (F = 38.444, df= 2,57, *P*< 0.001). Caterpillars injected with PBS consumed significantly higher amount of leaf area compared with caterpillars that were infected with HdIV (both the treatments “PAR” and “CF”) (Supplementary figure 2). No significant differences in terms of leaf area consumed were found between parasitized caterpillars and those injected with calyx fluid containing HdIV particles (Supplementary figure 2).

**Supplementary Figure 3.** Amount of leaf area consumed by L3 *Spodoptera frugiperda* caterpillars after feeding on corn plants for 24 hours. Plants were exposed to the following treatments: (PBS) caterpillars injected with phosphate-buffered saline; (PAR) caterpillars parasitized by *Hyposoter didymator*; (CF) caterpillars injected with calix fluid (containing virions) isolated from the parasitoid *H.* *didymator*. Different letters above bars indicate significant differences among treatments (ANOVA, P < 0.05)***.***


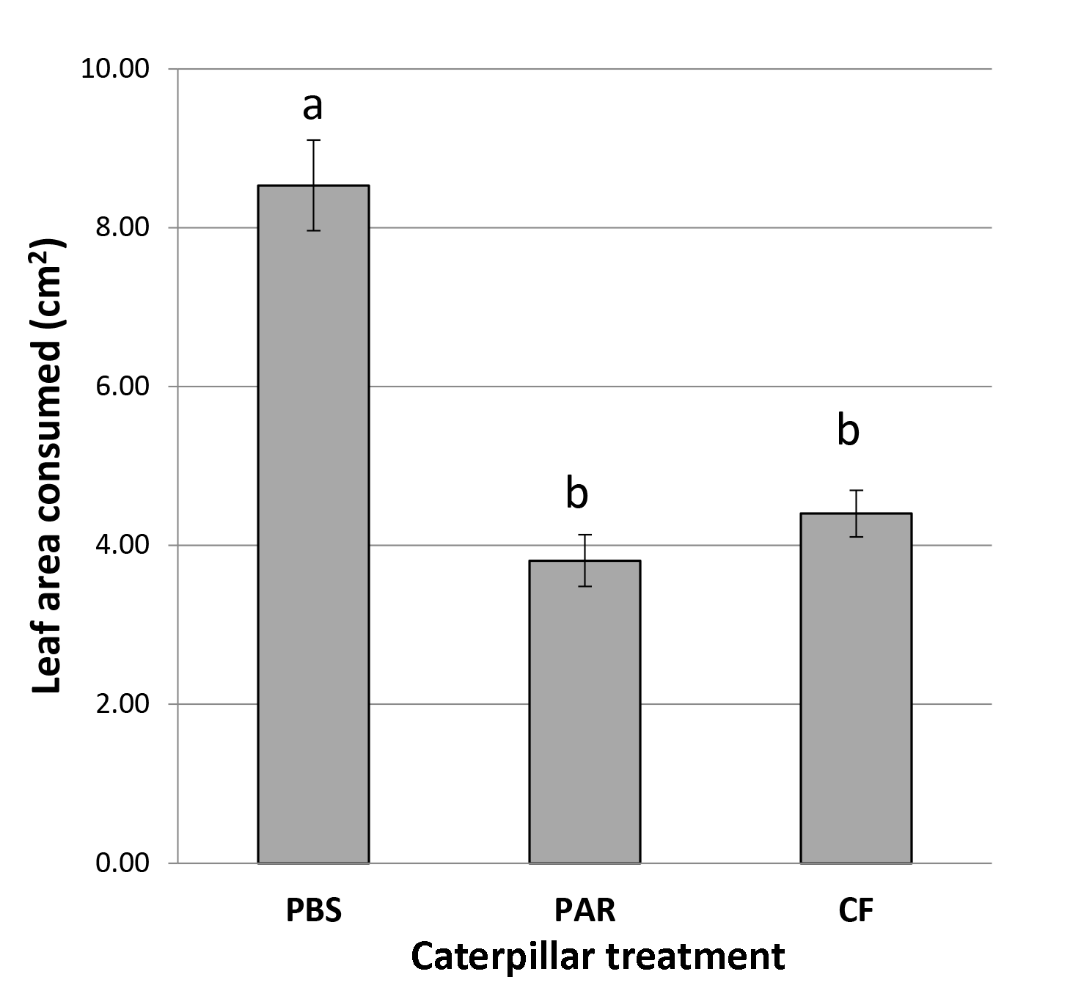

Supplement: Supplementary file 1 — Supplementary Material [file MEC-30-4567-s003.docx]
